# Day-by-Day Distribution of SARS-CoV-2 RT-PCR Cycle Threshold Values in Outpatient Care: Associations with Symptom Onset and Fever Severity

**DOI:** 10.3390/diagnostics16081118

**Published:** 2026-04-08

**Authors:** Masamichi Yoshika

**Affiliations:** Yoshika Clinic, 3-14-4 Daido Minami, Higashi Yodogawa-Ku, Osaka 533-0012, Japan; masa@yoshika-clinic.com

**Keywords:** SARS-CoV-2, RT-PCR, cycle threshold, symptom onset, fever, primary care

## Abstract

**Background/Objectives**: Cycle threshold (Ct) values from SARS-CoV-2 RT-PCR are widely reported in clinical practice, yet their interpretation in outpatient settings remains challenging due to substantial temporal and clinical variability. This study aimed to characterize day-by-day Ct distributions after symptom onset and to evaluate how symptom timing and fever severity inform diagnostic interpretation in primary care. **Methods**: We conducted a single-center retrospective study of 906 outpatients with COVID-19 who underwent saliva RT-PCR testing (January 2022–April 2023). Ct values were summarized according to days since symptom onset (Day 0–14). Peak self-reported temperature was categorized into 1 °C strata (36–40 °C), with temperature analyses restricted to patients tested on Day 0. Spearman’s correlation and multivariable linear regression with 95% confidence intervals were used to assess associations. **Results**: Ct values increased with longer intervals from symptom onset but demonstrated substantial variability within each day (Spearman’s ρ = 0.166, *p* < 0.001). On Day 0, higher temperature strata were associated with lower Ct values (*p* = 0.018). In multivariable analysis, days since onset, temperature category, sex, and age group were independently associated with Ct values, whereas vaccination doses and comorbidities were not. **Conclusions**: Incorporating symptom onset timing and fever severity may support more nuanced, context-based interpretation of Ct values in primary care, rather than reliance on isolated thresholds.

## 1. Introduction

Cycle threshold (Ct) values obtained using real-time reverse transcription polymerase chain reaction (RT-PCR) testing for coronavirus disease 2019 (COVID-19) are routinely reported in clinical practice and are commonly interpreted as indirect indicators of viral RNA burden. Ct values are inversely correlated with viral quantity, and associations with viral culture positivity, disease severity, and clinical outcomes have been reported [1,2,3,4,5,6]. Despite known variability related to assay platforms, specimen types, and pre-analytical conditions, Ct values are frequently referenced in clinical discussions [1,2,3,4,5,6,7].

From a virological perspective, viral RNA levels typically peak around the time of symptom onset and decline thereafter. Several studies have demonstrated positive associations between days since symptom onset and Ct values [8,9]. However, most prior reports summarize Ct values using aggregated statistics or broad time windows. Detailed day-by-day descriptions of Ct value distributions after symptom onset remain limited, particularly in routine outpatient settings where RT-PCR testing is widely performed and Ct values are interpreted without precise temporal context.

Fever is a representative early manifestation of COVID-19 and reflects innate immune activation mediated by inflammatory cytokines such as interleukin-1, interleukin-6, and tumor necrosis factor-α [10]. Because fever often coincides with early viral replication, body temperature at symptom onset may provide clinically accessible context for interpreting early Ct values. Nevertheless, few studies have examined Ct value distributions stratified by body temperature categories in relation to exact timing from symptom onset, especially in primary care populations.

In addition, host characteristics—including age, sex, comorbidities, and vaccination status—may influence viral kinetics and clinical presentation. Simultaneous evaluation of these factors using multivariable approaches is necessary to understand the real-world variability of Ct values reported in outpatient practice.

Accordingly, this study aimed (1) to describe day-by-day distributions of RT-PCR Ct values according to days since symptom onset; (2) to examine the association between body temperature categories and Ct values on the day of symptom onset; (3) to identify factors independently associated with Ct values and body temperature categories using multivariable analyses. By presenting Ct values as day-specific distributions, we sought to provide reference data to support contextual interpretation of outpatient RT-PCR results in clinical virology practice.

## 2. Materials and Methods

### 2.1. Study Design and Participants

This single-center, retrospective observational study was conducted at a primary care fever outpatient clinic. Consecutive patients who visited the clinic between January 2022 and April 2023 and were diagnosed with COVID-19 using real-time RT-PCR were eligible for inclusion.

A total of 906 outpatients were included in the analysis. All RT-PCR tests were performed using saliva specimens collected at the time of the outpatient visit, reflecting routine clinical practice in this setting.

### 2.2. Data Collection and Variable Definitions

Clinical and demographic data were extracted from electronic medical records, including age, sex, date of symptom onset, date of specimen collection, and RT-PCR Ct values. The number of days from symptom onset to specimen collection was calculated for each patient and used to define day-specific strata.

Body temperature was defined as the highest self-reported temperature after symptom onset, as recorded during the outpatient encounter. Because some patients had taken antipyretic agents or had defervesced by the time of presentation, this measure was used to approximate peak febrile response. Body temperature was categorized into five strata (36 °C to 40 °C in 1 °C increments).

Analyses focusing on “Day 0” were restricted to patients who underwent RT-PCR testing on the day of symptom onset.

Vaccination status was recorded as the total number of COVID-19 vaccine doses received (range, 0–5). Patients with unknown vaccination status were excluded from multivariable analyses. Information on comorbidities (including diabetes mellitus, hypertension, and cardiovascular disease) and prior SARS-CoV-2 infection history (self-reported or physician-diagnosed) was also collected.

### 2.3. RT-PCR Testing Procedures

Saliva specimens were collected using standardized sampling kits and processed according to the manufacturer’s instructions. RT-PCR testing was primarily outsourced to external laboratories using standardized equipment and reagents. Throughout the study period, the TaKaRa SARS-CoV-2 Direct Detection RT-qPCR Kit was used consistently.

A Ct value < 40 was defined as positive. Because all tests were performed using the same assay system, Ct values were analyzed without inter-laboratory normalization.

The assay targeted the SARS-CoV-2 N gene (N1/N2 regions) and an internal control (human RNaseP gene) according to the manufacturer’s instructions. Ct values analyzed in this study were those reported by the testing laboratory as part of routine clinical results. When multiple targets (e.g., N1 and N2 regions) were amplified, the specific algorithm used by the laboratory to determine the reported Ct value was not available to us.

### 2.4. Statistical Analysis

Continuous variables are presented as medians with interquartile ranges, and categorical variables as counts and proportions. The association between days since symptom onset and Ct values was assessed using Spearman’s rank correlation coefficient. Differences in Ct value distributions among groups were evaluated using the Kruskal–Wallis test, followed by Mann–Whitney U tests for pairwise comparisons where appropriate.

Multivariable linear regression analysis was performed with Ct value as the dependent variable. Explanatory variables included days since symptom onset, body temperature category, sex, age group (categorized by decades), number of vaccine doses, and presence of comorbidities. Sex was coded as 1 for male and 0 for female. Age group and body temperature category were treated as ordinal variables.

A separate multivariable linear regression model was constructed with body temperature category as the dependent variable, including Ct value, days since symptom onset, sex, age group, number of vaccine doses, and comorbidities as explanatory variables.

For multivariable analyses, complete-case analysis was performed, and 894 patients were included after excluding cases with missing data in one or more covariates. All statistical analyses were performed using Python (version 3.11) with the scipy, matplotlib, and statsmodels libraries. Regression coefficients are presented with 95% confidence intervals (CIs). A two-sided *p* value < 0.05 was considered statistically significant.

### 2.5. Ethical Considerations

This study was approved by the Ethics Committee of Yoshika Clinic (approval number: 0002). The study was conducted in accordance with the Declaration of Helsinki. All data were anonymized prior to analysis, and the requirement for written informed consent was waived due to the retrospective observational design in accordance with institutional guidelines.

## 3. Results

### 3.1. Patient Characteristics

A total of 906 outpatients were included in the analysis, comprising 493 females (54.4%) and 413 males (45.6%). The largest age group was individuals in their 20s, followed by those in their teens and 40s (Table 1).

Sex distribution across age groups did not differ significantly (χ^2^ = 16.66, df = 9, *p* = 0.054). Vaccination status differed significantly by age group (χ^2^ = 375.18, df = 45, *p* < 0.001), with a higher proportion of unvaccinated individuals among younger age groups and greater numbers of vaccine doses observed among older individuals.

Overall, 195 patients (21.5%) had at least one comorbidity (defined as the presence of one or more underlying conditions). The most common conditions were hypertension (*n* = 66, 7.3%), dyslipidemia (*n* = 42, 4.6%), diabetes mellitus (*n* = 19, 2.1%), and asthma (*n* = 25, 2.8%). Other comorbidities, including malignancy (*n* = 10), pregnancy (*n* = 6), and obesity (*n* = 5), were less frequent, while all remaining conditions were rare (≤3 cases each).

The distribution of comorbidities varied by age group and sex, with hypertension, dyslipidemia, and diabetes more frequently observed in middle-aged and older individuals. Detailed age- and sex-stratified distributions are provided in Supplementary Table S1.

### 3.2. Ct Values According to Days Since Symptom Onset

**Figure 1:**
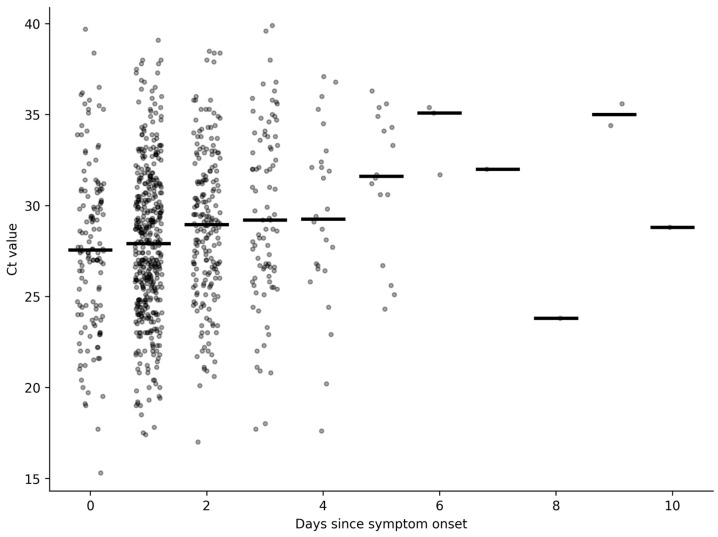
Distribution of Ct values according to days since symptom onset. Day-by-day distribution of SARS-CoV-2 RT-PCR Ct values according to days since symptom onset. Scatter plot showing individual Ct values plotted against days since symptom onset (Day 0–10). Each dot represents one patient. Horizontal bars indicate median Ct values for each day. The number of cases per day decreases after Day 7.

The distribution of Ct values by days since symptom onset is shown in Figure 1.

From Day 0 to Day 10, Ct values demonstrated a gradual upward shift. The largest numbers of cases were observed on Day 0 (*n* = 152), Day 1 (*n* = 228), and Day 2 (*n* = 206). The number of observations decreased substantially after Day 7.

A weak but statistically significant positive correlation was observed between days since symptom onset and Ct values (Spearman’s ρ = 0.166, *p* < 0.001). Kruskal–Wallis testing demonstrated significant differences in Ct value distributions across days (*p* < 0.001). Wide interindividual variability was observed within each day.

### 3.3. Ct Values by Body Temperature Category on the Day of Symptom Onset

**Figure 2:**
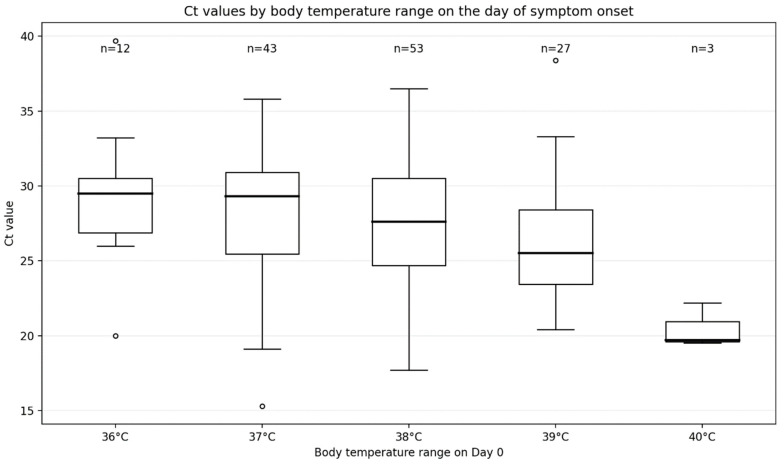
Ct values stratified by peak body temperature on the day of symptom onset. Distribution of SARS-CoV-2 RT-PCR Ct values by body temperature category on the day of symptom onset (Day 0). Box plots showing Ct value distributions stratified by body temperature categories (36–40 °C). Boxes represent the interquartile range (IQR), the central line indicates the median, and whiskers extend to 1.5 × IQR. Individual data points are overlaid. White circles represent outliers.

Ct value distributions stratified by body temperature category on the day of symptom onset (Day 0) are shown in Figure 2.

Ct values differed significantly across temperature categories (36–40 °C) (Kruskal–Wallis test, *p* = 0.018). Median Ct values were lowest in the highest temperature category (40 °C), with significant differences compared with the 36 °C and 37 °C categories (Mann–Whitney U test, *p* < 0.01).

No significant differences in Ct values by number of vaccine doses were observed on Day 0 (*p* = 0.311). On Day 2 after symptom onset, Ct value distributions did not differ significantly across temperature categories.

### 3.4. Body Temperature Category and Vaccination Status

The distribution of body temperature categories differed significantly according to the number of vaccine doses received (χ^2^ = 60.82, df = 25, *p* < 0.001). Higher temperature categories were less frequent among individuals with a greater number of vaccine doses.

### 3.5. Factors Associated with Ct Values

Results of the multivariable linear regression analysis with Ct value as the dependent variable are shown in Table 2.

Days since symptom onset (β = 0.683, 95% CI: 0.457–0.909; *p* < 0.001), body temperature category (β = −0.348, 95% CI: −0.636 to −0.060; *p* = 0.018), male sex (β = −1.153, 95% CI: −1.705 to −0.601; *p* < 0.001), and age group (β = −0.040, 95% CI: −0.060 to −0.021; *p* < 0.001) were independently associated with Ct values. Vaccination dose number and comorbidities were not significantly associated. The model demonstrated modest explanatory power (R^2^ = 0.087).

### 3.6. Factors Associated with Body Temperature Category

Multivariable linear regression analysis with body temperature category as the dependent variable is shown in Table 3.

Ct value (β = −0.018, 95% CI: −0.033 to −0.003; *p* = 0.018), age group (β = −0.011, 95% CI: −0.016 to −0.007; *p* < 0.001), and number of vaccine doses (β = −0.060, 95% CI: −0.115 to −0.006; *p* = 0.030) were independently associated with body temperature category. The model explained a limited proportion of variance (R^2^ = 0.075).

## 4. Discussion

In this real-world outpatient study, we organized SARS-CoV-2 RT-PCR Ct values as day-by-day distributions from symptom onset and examined their relationship with routinely available clinical features, particularly body temperature at onset. Rather than proposing Ct values as standalone indicators of infectivity or clinical decision-making, this analysis aimed to provide a practical reference framework for interpreting outpatient RT-PCR results within their temporal and clinical context. The marked variability observed both across and within individual days highlights the limitations of single-value interpretation and supports a distribution-based approach in routine clinical virology practice [6,11,12].

Ct values showed a gradual upward shift with increasing days since symptom onset, consistent with established viral RNA kinetics [4,8,9]. However, the observed correlation was weak, and substantial interindividual variability was evident even among patients tested on the same day. This weak correlation indicates that the timing of testing alone has limited predictive value for individual Ct values. From a diagnostic standpoint, this finding indicates that timing alone is insufficient to contextualize Ct values at the individual level. Presenting Ct values as day-specific distributions, rather than relying solely on summary statistics or fixed thresholds, more closely reflects how RT-PCR results are encountered and interpreted in outpatient care [6,12].

On the day of symptom onset, higher body temperature categories were associated with lower Ct values. Fever reflects early host inflammatory responses mediated by cytokines such as interleukin-1, interleukin-6, and tumor necrosis factor-α and may partially coincide with early viral replication [10]. This association was most apparent on Day 0 and was not observed on later days, suggesting a time-dependent relationship. Importantly, this finding should not be interpreted as supporting the use of fever to infer infectivity or transmission risk. Rather, it suggests that simple clinical metadata, such as body temperature at presentation, may assist in contextualizing early Ct values when testing is performed close to symptom onset. However, this association was modest and should be interpreted cautiously.

Multivariable analyses demonstrated that days since symptom onset, body temperature category, sex, and age group were independently associated with Ct values, whereas vaccination status and comorbidities were not. Previous studies have reported heterogeneous associations between Ct values, vaccination, and host factors depending on study design and circulating variants [13,14,15,16,17]. The explanatory power of the regression models was low (R^2^ = 0.087 and 0.075), indicating that these variables account for only a small proportion of Ct value variability. This further highlights the inherent variability of Ct values and reinforces that they should not be interpreted as precise individual-level predictors. The modest explanatory power of our models underscores that Ct values should be interpreted probabilistically and within context, rather than as precise quantitative surrogates of viral burden [6].

This suggests that multiple unmeasured factors may contribute to Ct variability, including differences in specimen collection (e.g., saliva volume and timing), host immune response, viral factors such as circulating variants, and pre-analytical conditions such as recent food intake or oral hygiene. Several methodological considerations merit attention. Although saliva-based RT-PCR has demonstrated a comparable diagnostic performance to nasopharyngeal swabs in appropriate contexts [18,19], Ct values are influenced by specimen type, assay design, amplification efficiency, and laboratory platforms [20,21,22,23]. In this study, saliva specimens were used consistently, reflecting routine outpatient practice and offering practical advantages. Although saliva-based RT-PCR has demonstrated comparable diagnostic performance to nasopharyngeal swabs in appropriate contexts [18,19], absolute Ct values should not be directly compared across different specimen types or assay systems. In addition, saliva samples may exhibit greater variability in viral RNA concentration compared with nasopharyngeal swabs, which could contribute to the wide distribution of Ct values observed within each day. In addition, viral variants circulating during the study period may have influenced Ct distributions [13,24]. Accordingly, our findings should be interpreted as assay- and context-specific reference data rather than universally applicable thresholds.

This study has several limitations. It was conducted at a single primary care clinic and included predominantly mild outpatient cases, limiting generalizability. Body temperature was based on self-reported peak values rather than standardized measurements at the time of sampling, introducing potential recall bias. This approach may partially mitigate the impact of antipyretic use; however, residual bias cannot be excluded. Furthermore, some patients may have used antipyretic medications prior to presentation, which could have affected the recorded body temperature and attenuated the observed associations. However, detailed data on antipyretic use were not consistently available, and this potential bias should be considered when interpreting the findings. Viral culture, genomic sequencing, and direct measures of transmission were not assessed; therefore, this study does not address infectivity or guide infection control decisions [1,6]. We did not have access to detailed target-specific amplification data, and Ct values were analyzed as reported by the external laboratory. These limitations reflect the realities of routine outpatient practice, where Ct values are most frequently interpreted.

In summary, by organizing SARS-CoV-2 RT-PCR Ct values as day-by-day distributions and examining their relationship with fever at symptom onset, this study provides a pragmatic reference framework for interpreting outpatient RT-PCR results. Given the relatively weak statistical associations and low explanatory power, these findings should be interpreted as indicative of general trends rather than precise predictive relationships. The findings emphasize variability rather than deterministic prediction and support a contextual, distribution-based approach to Ct value interpretation using simple clinical metadata available at presentation. Such reference data may complement laboratory reporting and assist clinicians and laboratorians in situating individual Ct results within their appropriate temporal and clinical context without overstating their diagnostic or prognostic implications.

These findings support a contextual and distribution-based approach to interpreting SARS-CoV-2 RT-PCR Ct values in outpatient practice and may assist clinicians in situating individual results within their appropriate temporal and clinical framework.

## Figures and Tables

**Table 1:** Baseline characteristics of the study population. (**A**) Distribution of Outpatient COVID-19 Patients by Age Group and Sex (*n* = 906). (**B**) COVID-19 Vaccination Status by Age Group and Sex (*n* = 906); Vaccination Status Was Unknown in 11 Patients.

**(A)**								
**Age Group**		**Female**			**Male**		**Total**	
0s		18			30		48	
10s		97			91		188	
20s		93			96		189	
30s		78			67		145	
40s		101			63		164	
50s		64			39		103	
60s		21			15		36	
70s		14			10		24	
80s		6			2		8	
90s		1			0		1	
**(B)**								
**Age Group**	**Sex**	**0 Doses**	**1 Dose**	**2 Doses**	**3 Doses**	**4 Doses**	**5 Doses**	**Unknown**
0s	Female	17	0	1	0	0	0	0
0s	Male	27	1	2	0	0	0	0
10s	Female	63	0	27	5	0	0	2
10s	Male	51	1	32	6	0	0	1
20s	Female	28	2	45	14	3	0	1
20s	Male	42	0	38	15	0	0	1
30s	Female	18	2	40	14	2	1	1
30s	Male	20	0	32	11	1	0	3
40s	Female	31	0	42	25	3	0	0
40s	Male	17	0	32	12	0	1	1
50s	Female	11	0	25	24	4	0	0
50s	Male	8	0	13	13	5	0	0
60s	Female	1	0	5	10	3	2	0
60s	Male	0	1	2	8	4	0	0
70s	Female	2	0	1	5	5	1	0
70s	Male	1	0	1	5	2	0	1
80s	Female	0	0	0	3	2	1	0
80s	Male	0	1	0	0	1	0	0
90s	Female	0	0	0	1	0	0	0

Abbreviations: 0s = 0–9 years; 10s = 10–19 years; 20s = 20–29 years; 30s = 30–39 years; 40s = 40–49 years; 50s = 50–59 years; 60s = 60–69 years; 70s = 70–79 years; 80s = 80–89 years; 90s = 90 years or older.

**Table 2:** Multivariable linear regression analysis for factors associated with SARS-CoV-2 RT-PCR Ct values.

Variable	β (95% CI)	p-Value
days since symptom onset	0.683 (0.457 to 0.909)	*p* < 0.001
body temperature category	−0.348 (−0.636 to −0.060)	*p* = 0.018
male sex	−1.153 (−1.705 to −0.601)	*p* < 0.001
age group (per decade)	−0.040 (−0.060 to −0.021)	*p* < 0.001
vaccine doses	−0.078 (−0.317 to 0.162)	*p* = 0.524
comorbidity	−0.620 (−1.441 to 0.201)	*p* = 0.139

Ct value was used as the dependent variable. Independent variables included days since symptom onset, body temperature category, sex, age group, number of vaccine doses, and presence of comorbidities. R^2^ = 0.087.

**Table 3:** Multivariable linear regression analysis for factors associated with body temperature category.

Variable	β (95% CI)	p-Value
days since symptom onset	−0.023 (−0.076 to 0.030)	*p* = 0.391
Ct	−0.018 (−0.033 to −0.003)	*p* = 0.018
male sex	0.092 (−0.034 to 0.219)	*p* = 0.153
age group (per decade)	−0.011 (−0.016 to −0.007)	*p* < 0.001
vaccine doses	−0.060 (−0.115 to −0.006)	*p* = 0.030
comorbidity	−0.008 (−0.195 to 0.180)	*p* = 0.936

Body temperature category was used as the dependent variable. Independent variables included Ct value, days since symptom onset, sex, age group, number of vaccine doses, and presence of comorbidities. R^2^ = 0.075.

## Data Availability

The datasets generated and analyzed during the current study are not publicly available due to institutional and ethical restrictions. The data may be made available from the corresponding author upon reasonable request, subject to approval by the institutional ethics committee.

## Supplementary Materials

The following supporting information can be downloaded at: https://www.mdpi.com/article/10.3390/diagnostics16081118/s1. Table S1: Age- and sex-stratified distribution of comorbidities among outpatient COVID-19 cases (*n* = 906).
