# Supplementary material for: Day-by-Day Distribution of SARS-CoV-2 RT-PCR Cycle Threshold Values in Outpatient Care: Associations with Symptom Onset and Fever Severity
Source: Diagnostics (Basel). 2026 Apr 8;16(8):1118. doi: 10.3390/diagnostics16081118 (PMC13114891; doi:10.3390/diagnostics16081118)
Supplement: Supplementary file 1 [file diagnostics-16-01118-s001.zip › diagnostics-4213173-supplementary.pdf]

Table S1. Age- and sex-stratified distribution of comorbidities among outpatient COVID-19 cases (n = 906).

Comorbidity	Total	<10 M	<10 F	10s M	10s F	20s M	20s F	30s M	30s F	40s M	40s F	50s M	50s F	60s M	60s F	70s M	70s F	80s M	80s F	≥90 M	≥90 F
Hypertension	66	0	0	0	0	2	0	1	1	9	5	5	11	6	10	6	6	2	1	0	1
Diabetes	19	0	0	0	0	0	1	0	1	0	1	3	2	3	3	2	1	2	0	0	0
Dyslipidemia	42	0	0	0	0	0	1	3	0	5	3	3	13	4	6	2	2	0	0	0	0
Asthma	25	0	1	1	0	3	4	3	1	4	3	0	4	0	1	0	0	0	0	0	0
Malignancy	10	0	0	0	0	0	0	0	0	0	3	0	5	0	1	0	1	0	0	0	0
Pregnancy	6	0	0	0	0	0	4	0	2	0	0	0	0	0	0	0	0	0	0	0	0
Obesity	5	0	0	0	0	1	0	2	0	1	0	1	0	0	0	0	0	0	0	0	0
Chronic kidney disease	2	0	0	0	0	0	0	0	0	1	0	1	0	0	0	0	0	0	0	0	0
Heart failure	2	0	0	0	0	0	0	1	0	0	0	1	0	0	0	0	0	0	0	0	0
Hyperuricemia	2	0	0	0	0	0	0	0	0	0	0	1	0	0	0	1	0	0	0	0	0
Chronic respiratory disease	2	0	0	0	0	1	0	0	0	0	0	1	0	0	0	0	0	0	0	0	0

Immunosuppression	3	0	0	0	0	1	0	0	0	1	0	0	0	0	0	0	0	1	0	0	0
Inflammatory bowel disease	2	0	0	0	0	0	0	0	0	2	0	0	0	0	0	0	0	0	0	0	0
Rheumatoid arthritis	2	0	0	0	0	0	0	0	0	0	0	0	1	0	0	1	0	0	0	0	0
Steroid use	1	0	0	0	0	0	0	0	0	0	0	0	1	0	0	0	0	0	0	0	0
Ischemic heart disease	1	0	0	0	0	0	0	0	0	0	0	0	0	1	0	0	0	0	0	0	0
Cerebral infarction	1	0	0	0	0	0	0	0	0	0	0	0	0	1	0	0	0	0	0	0	0
Sarcoidosis	1	0	0	0	0	0	0	0	0	0	0	1	0	0	0	0	0	0	0	0	0
Malignant lymphoma	1	0	0	0	0	0	0	0	0	0	0	0	0	0	0	1	0	0	0	0	0
Atrial fibrillation	1	0	0	0	0	0	0	0	0	0	0	0	0	0	0	0	0	0	1	0	0
Epilepsy	1	0	0	0	0	0	0	0	0	0	1	0	0	0	0	0	0	0	0	0	0

Abbreviations: F, female; M, male; <10 = under 10 years; 10s = 10–19 years; 20s = 20–29 years; 30s = 30–39 years; 40s = 40–49 years; 50s = 50–59 years; 60s = 60–69 years; 70s = 70–79 years; 80s = 80–89 years; ≥90 = 90 years or older.

Total indicates the sum across all age groups and sexes.
